# Fabrication of Ceftriaxone-Loaded Cellulose Acetate and Polyvinyl Alcohol Nanofibers and Their Antibacterial Evaluation

**DOI:** 10.3390/antibiotics11030352

**Published:** 2022-03-07

**Authors:** Youdhestar, Faraz Khan Mahar, Gotam Das, Ayesha Tajammul, Farooq Ahmed, Muzamil Khatri, Sheeraz Khan, Zeeshan Khatri

**Affiliations:** 1 Center of Excellence in Nanotechnology and Materials, Mehran University of Engineering and Technology, Jamshoro 76062, Pakistan; udhestar13te07@gmail.com (Y.); faraz13te91@gmail.com (F.K.M.); farooq.ahmed@faculty.muet.edu.pk (F.A.); sheerazkhan401@gmail.com (S.K.); 2 Department of Water Sanitation and Health Sciences, US-Pakistan Center for Advanced Studies in Water (USPCAS-W), Mehran University of Engineering and Technology, Jamshoro 76060, Pakistan; drayetalvi@gmail.com; 3 Department of Prosthodontics, College of Dentistry, King Khalid University, Abha 61421, Saudi Arabia; 4 Nano Fusion Technology Research Lab, Division of Frontier Fibers, Institute for Fiber Engineering (IFES), Interdisciplinary Cluster of Cutting-Edge Research (ICCER), Shishu University, Tokida 3-15-1, Ueda 386-8567, Japan; muzamilkhatri@gmail.com

**Keywords:** antibiotics, ceftriaxone, nanofibers, *E. coli*, *S. aureus*

## Abstract

Nanotechnology provides solutions by combining the fields of textiles and medicine to prevent infectious microbial spread. Our study aimed to evaluate the antimicrobial activity of nanofiber sheets incorporated with a well-known antibiotic, ceftriaxone. It is a third-generation antibiotic that belongs to the cephalosporin group. Different percentages (0, 5%, 10%, 15%, and 20%; based on polymer wt%) of ceftriaxone were incorporated with a polymer such as polyvinyl alcohol (PVA) via electrospinning to fabricate nanofiber sheets. The Kirby-Bauer method was used to evaluate the antimicrobial susceptibility of the nanofiber sheets using *Escherichia coli* (*E. coli*) and *Staphylococcus aureus* (*S. aureus*). For the characterization of the nanofiber sheets incorporated with the drug, several techniques were used, such as Fourier transform infrared spectroscopy (FTIR), X-ray diffraction (XRD), and scanning electron microscopy (SEM). Our results showed that the nanofiber sheets containing ceftriaxone had potential inhibitory activity against *E. coli* and *S. aureus* as they had inhibition zones of approximately 20–25 mm on Mueller-Hinton-agar-containing plates. In conclusion, our nanofiber sheets fabricated with ceftriaxone have potential inhibitory effects against bacteria and can be used as a dressing to treat wounds in hospitals and for other biomedical applications.

## 1. Introduction

The appearance and extent of lethal diseases caused by viruses and bacteria are a serious challenge of public health and safety, despite the medical system improving over the 20th and 21st centuries [1]. Antibacterial activity relates to compounds that locally kill bacteria or slow down their growth, without being generally toxic to surrounding tissue. Currently, most antibacterial agents are chemically modified natural compounds, such as b-lactams (e.g., penicillins), cephalosporins, and carbapenems [2]. In general, agents are classified as either bactericidal, which kill bacteria, or bacteriostatic, which slow down bacterial growth. Antibacterial agents are paramount to fight infectious diseases [3,4]. Electrospinning has been used to manufacture multifunctional materials with antibacterial, antiviral, and nontoxic qualities into nanofibers without losing their intrinsic capabilities, paving the way for novel ways to prepare surgical goods such as gowns and face masks [5,6,7]. Nanotechnology plays a critical role in the development of antimicrobial products [8,9]. Due to their advantages of having a large specific surface area, being lightweight and flexible, and having high stability and good air permeability, nanofibers are widely used in biomedical materials [10], filtration, protection [11,12], catalysis [13], and water treatment [14,15]. Infiltration and protection applications, nanofibers provide better air permeability, higher drug-loading capacity, and greater packaging efficiency, making them the first choice for loading antibacterial and antiviral drugs and opening a large market for antibacterial and antiviral protective equipment materials [16]. Drawing, template synthesis, phase separation, self-assembly, and electrospinning techniques are some of the approaches that are used to create these nanofibers [17]. Several researchers have investigated and developed electrospun nanofibers with antibacterial properties. These nanofibers were loaded with a variety of elements, including nanoparticles, medicines, and herbal extracts [18]. Ashraf et al. used the electrospinning technique to create silver-loaded cellulose acetate nanofibers [19]. Ahir et al. carried out a similar study, in which they used PEO and poly-D,L-lactide electrospun nanofibers to load copper nanoparticles. After 48 h of interaction with bacteria, copper-loaded nanofibers demonstrated a 41% reduction in *P. aeruginous* bacteria and a 50% reduction in *S. aureus* bacteria [20]. Malwal et al. synthesized CuO-ZnO-loaded polyvinyl alcohol electrospun nanofibers and tested their antibacterial efficacy against *E. coli* and *S. aureus* bacteria. They discovered that CuO-ZnO-loaded electrospun nanofibers with a concentration of 450 g/mL significantly inhibited *E. coli* bacteria and *S. aureus* growth [21].

Various investigations have been carried out on the production of antibacterial nanofibers using electrospun nanofibers loaded with various medicines. Ajmal et al. created electrospun poly (lactic-co-glycolic acid)-alginate nanofibers and subsequently loaded the ciprofloxacin drug with these electrospun nanofibers. They achieved this by utilizing the broth microdilution method and determining ciprofloxacin’s MIC value against *S. aureus* bacteria [22].

Ceftriaxone is a commonly used, third-generation active antibiotic agent against many Gram-positive and Gram-negative pathogens, such as *Escherichia coli* and *Staphylococcus aureus*, and it has been reported with various materials such as zinc oxide, silver nanoparticles, copper salt, and chitosan nanoparticles [23,24,25,26,27]. However, the application of ceftriaxone in nanofibers has only been reported in PVA nanofibers [28]. Due to the high potential of ceftriaxone, there is a need to further explore its antibacterial applications with different nanofibers. Moreover, cellulose acetate (CA) is a preferred polymer for producing different materials. Electrospun CA nanofibers have been used in medical applications due to their remarkable properties including biocompatibility, water insolubility, good mechanical properties, low toxicity, and excellent chemical resistance [29,30]. Therefore, we fabricated ceftriaxone-loaded CA and PVA nanofibers and compared their results. The electrospinning technique was employed to create ceftriaxone-loaded PVA (PVA/CEF) and ceftriaxone-loaded CA (CA/CEF) nanofibers in this study. For the creation of electrospun nanofibers, four different concentrations of ceftriaxone were utilized. The antimicrobial activity of the produced nanofibers was tested against *E. coli* and *S. aureus* bacteria and showed prominent results.

## 2. Experimental Procedure

### 2.1. Materials

Cellulose acetate (CA) was purchased from Sigma Aldrich (St. Louis, MO, USA) (Mw 30 K). Polyvinyl alcohol (PVA) polymer (Mw 89,000–98,000) was purchased from Sigma Aldrich (St. Louis, MO, USA). Ceftriaxone was obtained from Macter International Limited, Karachi.

### 2.2. Method

#### 2.2.1. Preparation of Nanofibers

Cellulose acetate (CA) solution of 18% was prepared in a solvent mixture of acetone and DMF with a ratio 2:1 and was kept on magnetic stirring for 24 h. After complete dissolution of CA polymer, different concentrations of ceftriaxone (0, 5%, 10%, 15%, and 20%; based on polymer wt%) were added. Then, stirring continued for 30 min to form a homogeneous solution. The prepared solution was electrospun by filling it into a plastic syringe that was attached to a feed pump, setting the solution feed rate at 1 mL/h, setting the tip-to-collector distance at 12 cm, and supplying a 12.5 kV power for the formation of ceftriaxone-loaded CA nanofibers.

Similarly, 10% PVA solution was prepared by mild stirring at room temperature. After the complete dissolution of PVA polymer, different concentrations of ceftriaxone (0, 5%, 10%, 15%, and 20%; based on polymer wt%) were added. Then, stirring continued for 30 min to form a homogeneous solution. The prepared solution was electrospun at 16 kV applied voltage, feed rate was 1 mL/h, and the distance between syringe tip to collector was set at 10 cm. The prepared ceftriaxone-loaded PVA nanofiber membranes were dried for 24 h at room temperature.

#### 2.2.2. Antimicrobial Activity of Nanofibers

The disc diffusion method was used to test the antibacterial activity against Staphylococcus aureus and Escherichia coli bacteria. The solutions were evaluated using a dilution method after *Escherichia coli* and *Staphylococcus aureus* were cultured in nutrient broth at 37 °C for 24 h. The number of viable bacterial cells was adjusted from 3 × 10^5^ cfu/mL to 4 × 10^5^ cfu/mL by serial dilution (four times) with 0.03 mol/L PBS. After the desired bacterial growth was reached, 0.03 g of nanofibers was placed into 65 mL of 0.3 mM PBS culture solution and 5 mL suspension of prepared bacterial solution. The flask was then agitated on a rotary shaker for 18 h at 150 rpm at 37.8 °C. The solution was diluted 10 times with 0.3 mM PBS, and this was repeated four times for a serial dilution. Finally, bacterial suspensions of various concentrations (1 mL each) were placed on an agar plate. The number of colonies grown on the agar plate was calculated by Equation (1) after 24 h of incubation at 37.8 °C:

(1) $$R=\frac{\left(B-A\right)}{B}\times 100\%$$

where ‘R’ denotes the percentage bacterial reduction, and ‘B’ and ‘A’ denote the number of live bacterial cells in the flasks of the treated and untreated samples, respectively, after shaking.

The Kirby Bauer method (disc diffusion method) was used to determine the antibacterial activity of PVA and CA nanofibers against *E.* *coli* and *S. aureus* pathogens. Each nanofiber sample of 6 mm diameter was mounted on Mueller-Hinton agar plates spread with bacterial colonies. Afterward, the Petri dishes were incubated for 48 h at 37 °C. After the incubation, the inhibition zones were measured and compared.

#### 2.2.3. Material Characterization

The physical morphology of neat CA, neat PVA, CA/CEF, and PVA/CEF nanofibers was assessed by scanning electron microscopy (SEM, JEOL JSM-6380L instrument, Tokyo, Japan). The chemical structure and changes were examined using IRPrestige-21 from Shimadzu (Kyoto, Japan) at ATR-FTIR mode at adsorption wavelengths between 500 and 4000 cm^−1^ at 25 °C. The crystalline and amorphous regions of the prepared nanofibers were observed by X-ray diffraction (XRD model D/max-HB, Rigaku).

## 3. Results and Discussion

### 3.1. Physical Morphology of Nanofibers

**Figure 1:**
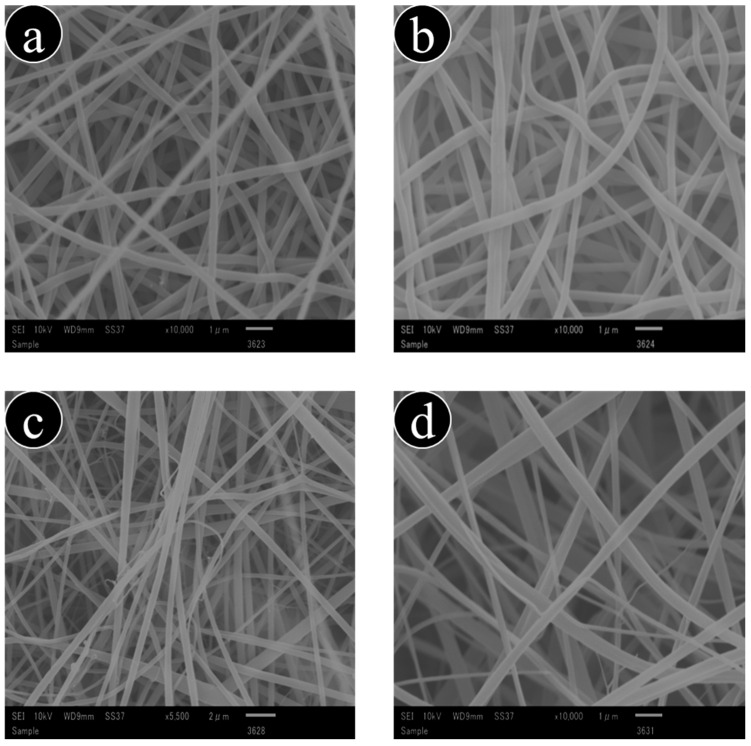
SEM images of nanofibers: (**a**) CA, (**b**) CA/Ceftriaxone, (**c**) PVA, and (**d**) PVA/Ceftriaxone.

Prepared nanofibers were characterized by SEM; the SEM images are shown in Figure 1a–d. The morphology of the nanofibers was described as smooth and beaded. The average diameter of pure CA nanofibers was 200 nm; after loading the ceftriaxone drug, the average diameter increased to 220 nm. Similarly, the average diameter of pure PVA nanofibers increased from 210 to 250 nm after loading the drug. After adding the ceftriaxone drug, the diameters of the nanofibers of both polymeric fibers increased. However, the smoothness of the nanofibers remained unaffected.

### 3.2. FTIR Spectra of Nanofibers

**Figure 2:**
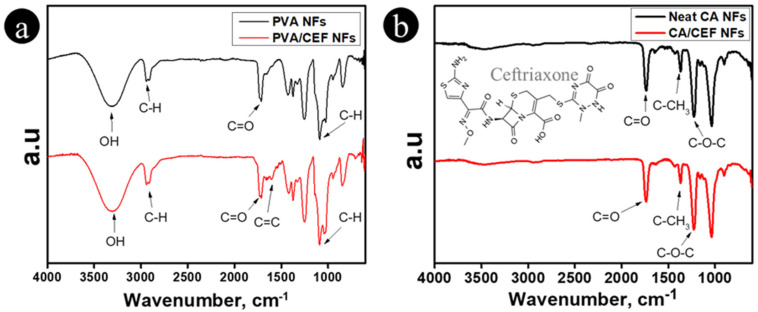
FTIR spectra of nanofibers: (**a**) PVA, PVA/CEF NFs, (**b**) CA, and CA/CEF NFs.

The nanofibers were characterized by FTIR to analyze their chemical structure and compatibility with ceftriaxone. As shown in Figure 2a, the FTIR spectra of PVA nanofibers and ceftriaxone-loaded PVA nanofibers showed major peaks at 3310, 2935, 1720, and 1084 cm^−1^, which corresponded to OH stretching, C-H stretch vibrations, C=O carbonyl stretching, and C-O-H stretching, respectively, in the PVA polymer [30]. After loading the ceftriaxone drug, a new peak was observed at 1603 cm^−1^, which corresponded to C=C stretching in the ceftriaxone drug [26,27,31]. The FTIR spectra of CA nanofibers (shown in Figure 2b) showed absorption peaks at 1736, 1372, and 1224 cm^−1^, indicating the presence of C=O, C-CH_3_, and C-O-C groups, respectively [32]. However, after loading the ceftriaxone drug, the obtained spectra did not show any new peaks, meaning that there was a physical interaction only.

### 3.3. XRD Spectra of Nanofibers

**Figure 3:**
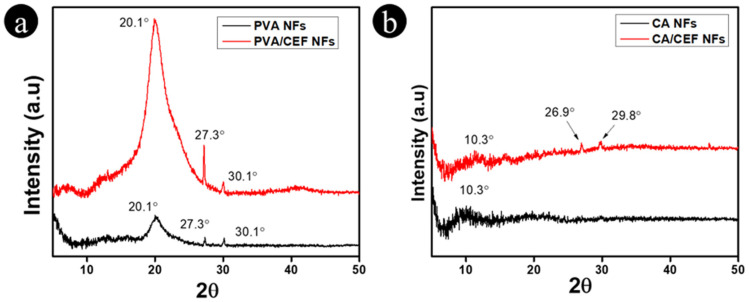
XRD spectra of nanofibers: (**a**) PVA and PVA/CEF NFs and (**b**) CA and CA/CEF NFs.

The XRD spectra of pure CA, pure PVA, ceftriaxone-loaded PVA (PVA/CEF), and ceftriaxone-loaded CA (CA/CEF) nanofibers were analyzed to assess the crystallinity of the nanofibers. The XRD spectra of pure PVA nanofibers, shown in Figure 3a, showed a typical broad peak at 20.1 that is generally associated with the amorphous structure of PVA and two small peaks at 27.3 and 30.1, indicating the crystallinity of the nanofibers [30]. After loading the ceftriaxone drug, the composite nanofibers showed similar spectra with enhanced peak intensities that indicated the crystalline nature of the drug in the nanofibers [26,33]. Figure 3b shows the XRD spectra of pure CA and CA/CEF nanofibers; the spectra of CA nanofibers showed a broad peak at 10 that indicated the amorphous polymeric nature of CA nanofibers [32,34]. The ceftriaxone-loaded CA nanofibers (CA/CEF) showed the same peak at 10 along with characteristic peaks at 26.9 and 29.8, which corresponded to the regular crystalline nature of the ceftriaxone drug in the nanofibers [26,33].

### 3.4. Antimicrobial Assessment of Nanofibers

**Figure 4:**
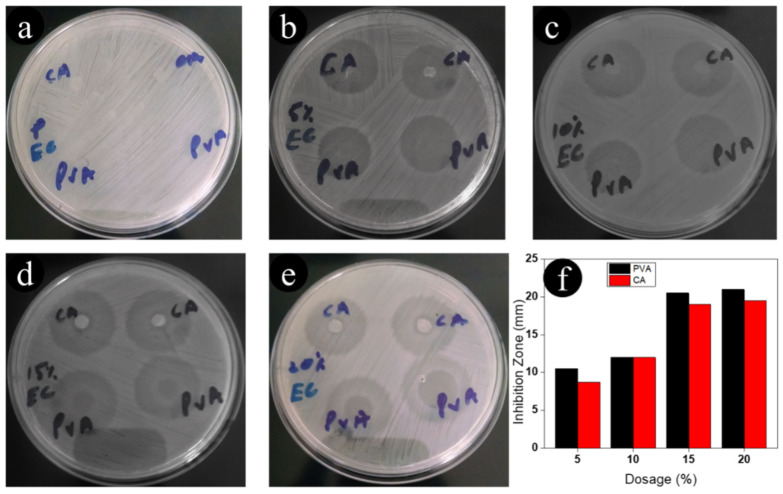
Antimicrobial assessment of CA and PVA nanofibers incorporated with different amounts of ceftriaxone, (**a**) 0, (**b**) 5%, (**c**) 10%, (**d**) 15%, and (**e**) 20% against *S. aureus*; (**f**) is a bar graph of the inhibition zones.

**Figure 5:**
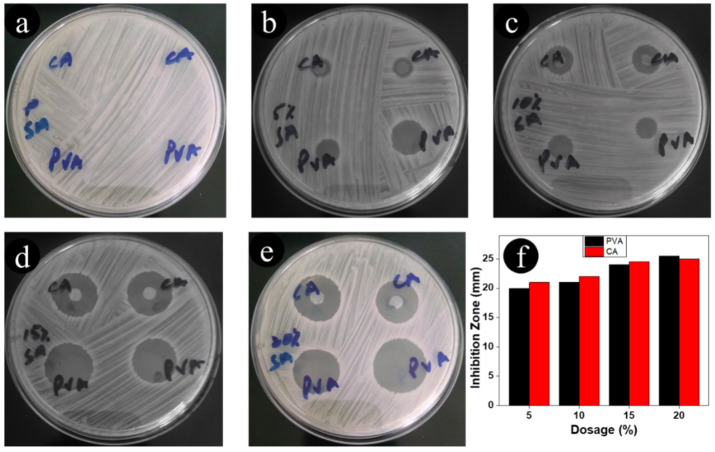
Antimicrobial assessment of CA and PVA nanofibers incorporated with different amounts of ceftriaxone, (**a**) 0, (**b**) 5%, (**c**) 10%, (**d**) 15%, and (**e**) 20% against *E. coli*; (**f**) is a bar graph of the inhibition zones.

The antimicrobial susceptibility of neat CA, neat PVA, and ceftriaxone-loaded nanofibers to *Staphylococcus aureus* (Gram-positive) microorganisms was evaluated. As shown in Figure 4, the antimicrobial activity images showed that the neat CA and neat PVA nanofibers had no inhibition zones against *Staphylococcus aureus*. When the amount of the ceftriaxone drug was increased from 5% to 20%, both CA and PVA nanofibers created an inhibition zone that had an approximate diameter of 25 mm against *Staphylococcus aureus*. Furthermore, the nanofibers containing 5% to 20% ceftriaxone created the same inhibition zone. It was concluded that a small amount (5%) of ceftriaxone in nanofibers was sufficient to create an inhibition zone. Both nanofibers were further evaluated for *E. coli* microorganisms, and the results are presented in Figure 5a–e. The results revealed that neat CA and neat PVA nanofibers did not possess an inhibition effect. However, the nanofibers loaded with ceftriaxone showed an inhibition zone of approximately 20 mm against *E. coli.* In the case of *E. coli*, the inhibition zone increased with an increasing amount of loaded drug. We found that 15% loaded drug was sufficient to create an inhibition zone against *E. coli*.

## 4. Conclusions

The nanofiber composites of PVA/CEF and CA/CEF were successfully prepared by the electrospinning technique, which was followed by an antibacterial assessment. The ceftriaxone drug was loaded into nanofiber sat different concentrations (0, 5%, 10%, and 20%). The produced nanofibers were smooth and beaded and had a diameter of 220 nm. The nanofibers showed a good inhibition zone against pathogens. PVA/CEF nanofibers showed inhibition zones of 20 and 25 mm against *E. coli* and *S. aureus*, respectively. CA/CEF nanofibers created inhibition zones of 20 and 25 mm against *E. coli* and *S. aureus*, respectively. The produced nanofiber composites maybe used in antibacterial applications for different medical purposes.

## Data Availability

The data can be requested from the corresponding author of the article.
